# Neural Mechanisms That Underlie Angina-Induced Referred Pain in the Trigeminal Nerve Territory: A c-Fos Study in Rats

**DOI:** 10.1155/2013/671503

**Published:** 2013-07-28

**Authors:** Bunsho Hayashi, Masako Maeda, Masayoshi Tsuruoka, Tomio Inoue

**Affiliations:** ^1^Department of Physiology, Showa University School of Dentistry, 1-5-8 Hatanodai, Shinagawa-ku, Tokyo 142-8555, Japan; ^2^Department of Physical Therapy, Teikyo Heisei University Faculty of Community Health Care, 4-1 Uruido Minami, Ichihara, Chiba 290-0193, Japan

## Abstract

The present study was designed to determine whether the trigeminal sensory nuclear complex (TSNC) is involved in angina-induced referred pain in the trigeminal nerve territory and to identify the peripheral nerve conducting nociceptive signals that are input into the TSNC. Following application of the pain producing substance (PPS) infusion, the number of Fos-labeled cells increased significantly in the subnucleus caudalis (Sp5C) compared with other nuclei in the TSNC. The Fos-labeled cells in the Sp5C disappeared when the left and right cervical vagus nerves were sectioned. Lesion of the C1-C2 spinal segments did not reduce the number of Fos-labeled cells. These results suggest that the nociceptive signals that conduct vagal afferent fibers from the cardiac region are input into the Sp5C and then projected to the thalamus.

## 1. Introduction

Angina pectoris has been recognized as the cause of a variety of cardiac symptoms, including chest pain and pain that may radiate either to arm or to the neck and jaw. Foreman and colleagues anesthetized a primate and recorded the responses from the spinothalamic tract (STT) cells in the T1–T5 and C5-C6 segments of the animal's spinal cord following either occlusion of the coronary artery or the injection of algesic chemicals into the pericardial sac. The authors also found a convergence of visceral and somatic input to the chest and upper arm [1]. Their findings were consistent with the observation that referred pain associated with an attack of angina pectoris commonly occurs in proximal somatic fields. The referred pain is explained by the convergence of visceral and somatic inputs to the same dorsal horn neuron in the nociceptive ascending pathway [2]. 

 It is well known that angina pectoris induces toothache or neck and jaw pain. The dental literature has described the presence of toothaches attributed to angina attacks and coronary artery disease [3]. Thus, referral to the somatic structures in the territory of the trigeminal nerve is one of the characteristics of anginal pain, and the neural mechanisms of this pain are of particular interest because such mechanisms appear to be due to a convergence of trigeminal and spinal inputs in either the spinal cord or the trigeminal sensory nucleus. Several lines of electrophysiological studies have demonstrated that an injection of algesic chemicals into the pericardial sac results in increased activity in C1-C2 STT cells [4–7]. This finding suggests that angina-induced referred pain in the trigeminal nerve territory is attributed to the convergence of both inputs from the trigeminal nerve territory and inputs from the cardiac region to C1-C2 STT cells because it is known that these cells receive converging somatic information from the neck and jaw regions [8]. However, angina-induced referred pain in the trigeminal nerve territory induced by the convergence of both inputs from the cardiac region and inputs from the neck and jaw regions to C1-C2 STT cells was not found in clinical or experimental observations.

 The trigeminal sensory nuclear complex (TSNC) is one of the components of the ascending sensory pathway that transmits sensory signals from the orofacial region to the higher centers of the brain [9]. Therefore, this complex may be considered to a candidate for the relay nuclei that transmits angina-induced referred pain. It is of particular interest to examine such a possibility because the mechanisms appear to be a convergence of trigeminal and spinal inputs in the TSNC. One of the objectives of the present study was to determine whether the TSNC is involved in the ascending sensory pathway that transmits nociceptive signals from the cardiac region to the thalamus in the brain.

During the twentieth century, it was believed that nociceptive information from the cardiac region was input to the thoracic segments of the spinal cord via the cardiac sympathetic nerve, while the vagal afferent fibers were thought to transmit innocuous cardiac sensory information [10–13]. Several morphological and electrophysiological studies from the late 1990s showed that vagal afferent fibers and the nucleus tractus solitarius play an important role in angina-related referred pain in the trigeminal nerve territory [4, 5, 7, 14]. This finding is consistent with clinical observations that neck and jaw pain continues or develops after the use of a sympathectomy to relieve anginal pain [15, 16]. The second aim of the present study was to identify the peripheral nerve that conducts the nociceptive signals that are input into the TSNC. 

## 2. Materials and Methods

All of the experimental protocols used in the present study were approved by the Institutional Animal Care and Use Committee of Showa University and were in accordance with the guideline of the International Association for the Study of Pain [17]. For example, the number of animals was kept to a minimum to minimize the animal's discomfort. 

### 2.1. Animals

The experiments were performed on male Wistar rats weighing 400–550 g (*n* = 30, Charles River, Tokyo). The animals were housed in groups of 3-4 in a cage containing sawdust bedding and were granted free access to rat chow and water. The laboratory was equipped with a 12 h/12 h (8 a.m./8 p.m.) light-dark cycle. Room temperature and humidity were maintained at 23.5°C and 60%, respectively. 

### 2.2. Surgical Preparation

The animals were anesthetized with urethane (1.2 g/kg i.p.). After a tracheotomy was performed, the animals were artificially ventilated to maintain an end-tidal CO_2_ level between 3.5 and 4.5%. An adequate level of anesthesia was determined by the absence of pupillary dilation or increases in heart rate. Under the anesthetic condition in the present study, no spontaneous movement was observed in the animals throughout the experiments. Mean blood pressure was measured directly via a catheter that was inserted into the right femoral artery. Heart rate was monitored by electrocardiogram. The mean blood pressure and the electrocardiogram were fed into an A/D converter (CED 1401 +) and were recorded using Spike2 software (CED, Cambridge, UK). The animal's core body temperature was regulated at approximately 37.5°C using a thermostatically controlled heating blanket. 

### 2.3. Pain Producing Substance (PPS)

The cardiac nociceptive receptors were stimulated chemically using a modified pain producing chemical mixture that was designed to excite both the vagal and sympathetic afferent endings [18]. The PPS solution contained bradykinin (10^−5^ M), serotonin (10^−5^ M), prostaglandin E2 (10^−5^ M), histamine (10^−5^ M), and adenosine (10^−3^ M), all of which may be released during myocardial ischemia [1, 19, 20]. The chemicals were individually dissolved in saline solution to a concentration of 10^−3^ M and were kept frozen. On the day of the experiment, the stock solutions were warmed and further diluted in normal saline to concentrations of 10^−5^ M (except the adenosine which was diluted to a concentration of 10^−3^ M). 

### 2.4. Intrapericardial Infusion of the PPS Solution

Using a modification of the procedure that was described by Euchner-Wamser et al. [19], a catheter was placed in the pericardial sac. The catheter was made of silicone tubing (0.020 ID, 0.037 OD, 14–16 cm length) and was filled with 0.2–0.5 mL of warmed normal saline. A thoracotomy was performed on the left costal cartilages of ribs 1–3 to expose the thymus gland and the heart. The thymus gland was opened along the midline, and the catheter was carefully inserted into the pericardial sac over the left ventricle. The catheter was fixed in place by suturing together the two thymus lobes and the layers of the chest wall. To test the pericardial catheter, 0.2 mL of saline was easily injected and removed via a 1 mL syringe that was connected to the catheter.

For nociceptive chemical stimulation of the heart, 0.2 mL of the PPS solution was infused into the pericardial sac through the catheter over 20–30 s. The solution was then allowed to stand for 2 min. The PPS solution was withdrawn over 20–30 s, then 0.2 mL of saline flush was infused for 2 min to rinse away the chemicals within the pericardial sac. The infusion-withdrawal procedure of the PPS solution and saline flush comprised one set of the experimental protocol, and three sets of this procedure were repeated at an interval of 2-3 min as 1 process. Throughout the duration of the experiment, three processes were applied in total. At least 10 min elapsed between each process. At 1.5 h after the final infusion process of infusion, pontamine sky blue dye was infused into the pericardial sac to confirm that the PPS solution did not leak through to the outside of the pericardial sac. Saline was substituted for the PPS solution in the vehicle-infused group, although the experimental protocol remained identical to that performed on the PPS-infused group. In the untreated control group, the catheter was inserted into the pericardial sac but no solution was infused. 

### 2.5. Fos Study

At the end of each experiment, the animals were perfused intracardially with 0.02 M phosphate buffer (PBS), followed by 4% paraformaldehyde in 0.1 M phosphate-buffered saline (PBS). The spinal cord (C1-C2 segments) and the brain were removed and placed in 4% paraformaldehyde in 0.1 M phosphate-buffered saline (PH7.4) at 4°C for 3–7 days. The samples were then stored in a cryoprotected 30% sucrose solution in 0.1 M phosphate-buffered saline (PH 7.4) at 4°C for 3–7 days. Consecutive transverse 50 *μ*m sections were cut using a cryostat (IEC) at −20°C and then transferred immediately to ten alternate wells of polypropylene plates that contained PBS. The sections were washed three times in PBS for 10 min each and were then incubated in 3% H_2_O_2_ for 30 min to stop endogenous peroxidase activity. The sections were again washed three times for 10 min each in PBS. The sections were then incubated in a blocking solution that contained 3% normal goat serum (NGS) (Vertor Laboratories, USA) for 30 min. The sections were then incubated overnight in c-Fos antibody that was taken from rabbits (1 : 5000; #SC-52, Santa Cruz biotechnology, CA, USA) at 4°C. This incubation caused no cross-reactivity with the Jun protein (Oncogene). At the completion of this incubation, the sections were washed in cold PBS three times for 10 min each at room temperature and then incubated with biotinylated anti-rabbit IgG (1 : 1000, Dako) for 120 min and peroxidase-conjugated streptavidin (1 : 1000, Dako) for 60 min at room temperature. All of the antibodies were diluted with PBS, which contained 1% normal goat serum and 0.5% Triton X-100. For the peroxidase reaction, 3′,3′-diaminobenzidine tetrahydrochloride (DAB, Sigma) with nickel ammonium sulfate intensification was used. The sections were mounted on slides, air-dried overnight, and then counter-stained with neutral red when necessary. Finally, coverslips were applied to the slides.

### 2.6. Vagotomy and Spinal Cord Lesion

For the vagotomy, a tie was gently pulled around the nerve, and then the left and right cervical vagus nerves were separated from the carotid artery and cut with a scissors. In a second group of animals, the involvement of C1-C2 neurons in PPS-induced referred pain in the trigeminal nerve territory was examined by lesioning the C1-C2 segments of the spinal cord using a small knife without a laminectomy. After the lesion was performed, the exposed upper cervical enlargement of the spinal cord was covered with bone wax. The musculature and skin were sutured. The animals were provided at least 30 min rest after the procedure. 

### 2.7. Quantification and Statistical Analysis

For each animal, the Fos-labeled cell profiles in 110 serial sections (9.16–14.66 mm caudal to bregma) including the trigeminal brainstem sensory nuclear complex were counted at the light microscope level, and camera lucida drawings were made of the cells. The trigeminal sensory nuclear complex is divided into the principal sensory trigeminal nucleus (Pr5VL), subnucleus oralis (Sp5O), subnucleus interpolaris (Sp5I), and subnucleus caudalis (Sp5C). The nomenclature and nuclear boundaries were defined based on the atlas produced by Paxinos and Watson [21]. For each nucleus of the TSNC, the number of identified Fos-labeled cells was counted for every three sections, and the total number of Fos-labeled cells was calculated for each rat. 

The data are represented as the mean ± S.E.M. Statistical analyses were carried out using Student's paired *t*-test to compare single groups before and after treatment. The Fos data were analyzed using one-way ANOVA (the multiple comparison test) and/or Student's *t*-test, where applicable. A difference was accepted as significant when *P* < 0.05.

## 3. Results

The animal's mean blood pressure and heart rate after urethane anesthesia were approximately 80–100 mmHg and 300–350/min, respectively. To accomplish our objectives, special attention was given to the change in the mean blood pressure and the electrocardiogram following the infusion of either PPS solution or saline into the pericardial sac. No significant changes were observed in the mean blood pressure and the electrocardiogram for all of the rats that were tested (data not shown). Based on this finding, the influence of the infusion-withdrawal procedure of either PPS solution or saline flush into the pericardial sac could be excluded in the present study.

### 3.1. c-Fos Expression following the Infusion of PPS Solution into the Pericardial Sac

**Figure 1:**
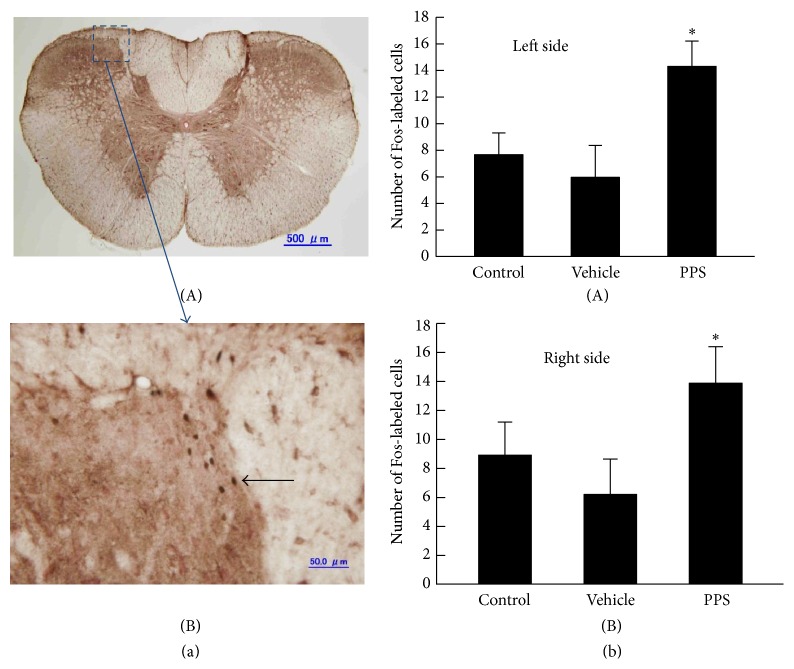
(a-A) Photomicrograph of c-Fos expression in the C2 spinal segment of a PPS-infused rat. The area bounded by the broken line is magnified 10 diameter (a-B). A black spot indicates a Fos-labeled cell (black arrow). (b) The number of Fos-labeled cells in uninfused control (Control, *n* = 4), vehicle-infused (Vehicle, *n* = 4), or PPS-infused (PPS, *n* = 4) rats. ^*^ *P* < 0.05, significantly different from the number of Fos-labeled cells in vehicle-infused rats.

Figure 1 shows the c-Fos expression in the C2 spinal segments following the infusion of the PPS solution into the pericardial sac. The number of Fos-labeled cells in the C2 spinal segment was significantly higher in the PPS-infused rats compared with the vehicle-infused and uninfused control rats. In the present study, data were used only from those animals whose number of Fos-labeled cells in the C2 spinal segment increased following the infusion of PPS solution into the pericardial sac because several lines of electrophysiological studies have demonstrated that an injection of algesic chemicals into the pericardial sac results in increased activity in C1-C2 STT cells [4–7]. 

**Figure 2:**
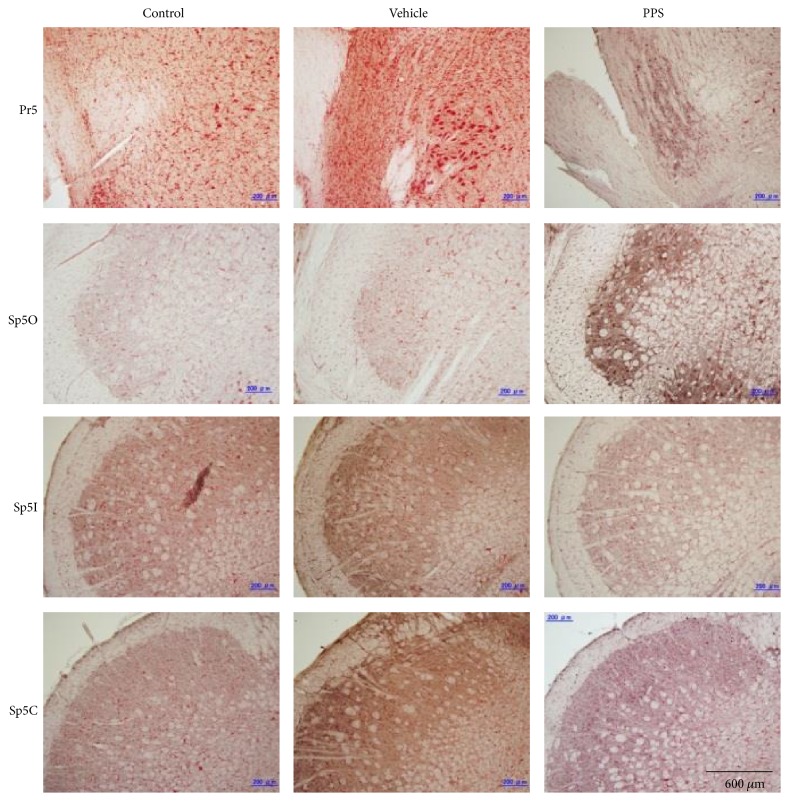
Photomicrographs of c-Fos expression in the left TSNC of uninfused control (Control), vehicle-infused (Vehicle), or PPS-infused (PPS) rats. Pr5, the principal sensory trigeminal nucleus; Sp5O, the subnucleus oralis; Sp5I, the subnucleus interpolaris; Sp5C, the subnucleus caudalis.

**Figure 3:**
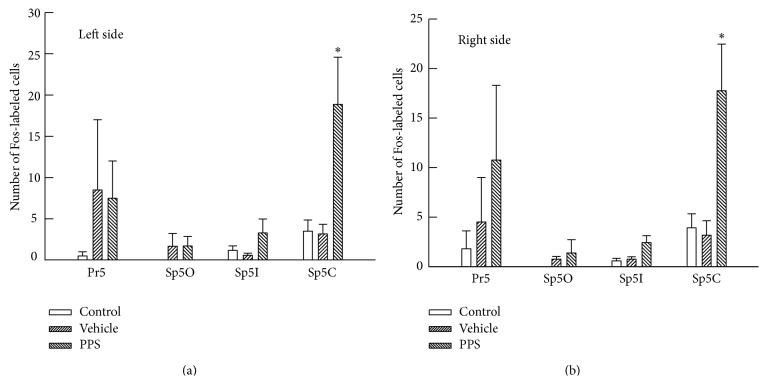
A comparison of the number of Fos-labeled cells across uninfused control (Control, *n* = 4), vehicle-infused (Vehicle, *n* = 4), and PPS-infused (PPS, *n* = 4) rats in the left (a) and right (b) TSNC. Pr5, the principal sensory trigeminal nucleus; Sp5O, the subnucleus oralis; Sp5I, the subnucleus interpolaris; Sp5C, the subnucleus caudalis. Note that a significant increase in the number of Fos-labeled cells was only observed in both sides of the Sp5C. ^*^ *P* < 0.05, significantly different from the number of Fos-labeled cells in vehicle-infused rats.

 In the TSNC, Fos-like immunoreactivity was observed in the PPS-infused rats (*n* = 4), in the vehicle-infused rats (*n* = 4), and even in the untreated control rats (*n* = 4). Examples of c-Fos expression are shown in Figure 2, and the number of Fos-labeled cells is summarized in Figure 3. The number of Fos-labeled cells for the Pr5, the Sp5O, and the Sp5I did not reveal significant differences across the treated groups. By contrast, for the Sp5C, a significant increase in the number of Fos-labeled cells was observed bilaterally in the PPS-infused rats. No difference was observed in the number of increased Fos-labeled cells in the PPS-infused rats between the left and right Sp5C (Figures 3(a) and 3(b)).

**Figure 4:**
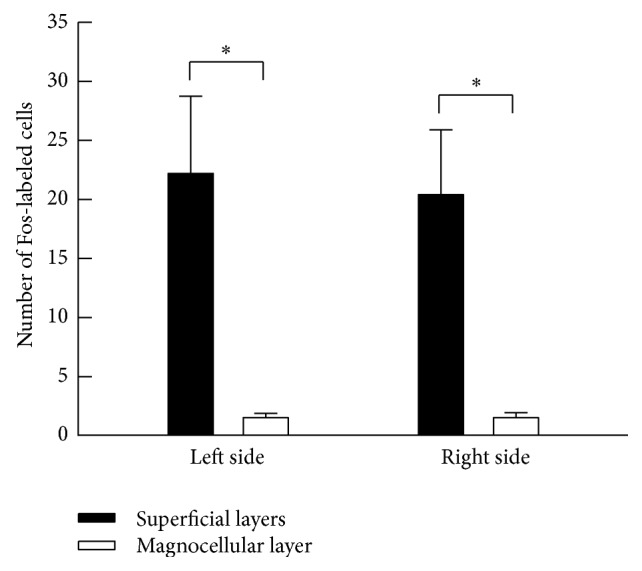
A comparison of the number of Fos-labeled cells between the superficial and the magnocellular layers in the Sp5C. Note that the number of Fos-labeled cells in the superficial layers was significantly higher than in the magnocellular layer. ^*^ *P* < 0.05, significantly different between two layers of the Sp5C.

A comparison of the number of Fos-labeled cells in the superficial layers (marginal layer and substantia gelatinosa) and magnocellular layer in the Sp5C indicated that the number of Fos-labeled cells in the superficial layers was significantly higher than in the magnocellular layer (Figure 4).

### 3.2. Effects of Vagotomy or C1-C2 Spinal Cord Lesion on c-Fos Expression

 Following either the vagotomy or a spinal cord lesion, the animal's mean blood pressure and heart rate did not significantly change compared to the values that were present before the surgical treatments, although they transiently increased after the lesion of the C1-C2 spinal segments. 

**Figure 5:**
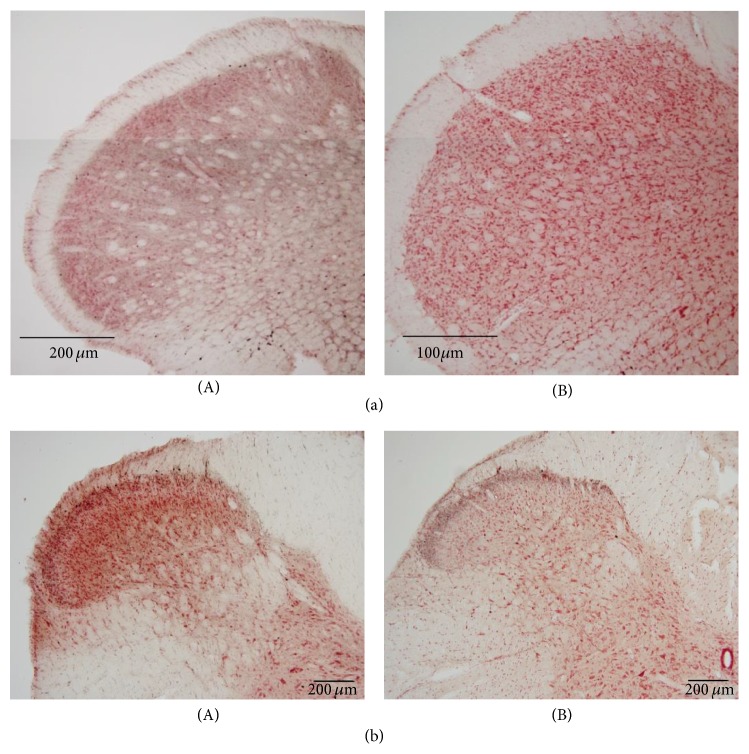
Effects of vagotomy on c-Fos expression in PPS-infused rats. The cervical vagus nerve was bilaterally sectioned. Photomicrographs show c-Fos expression in the left Sp5C (a) and the C2 spinal segment (b). Fos-labeled cells were observed in intact rats (a-A and b-A). The Fos-labeled cells were not observed in the vagotomy rats (a-B and b-B).

**Figure 6:**
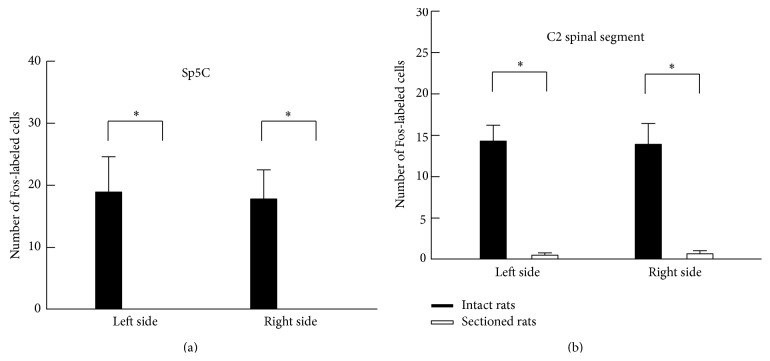
A comparison of the number of Fos-labeled cells between the intact and vagotomy rats following an infusion of PPS solution. Note that c-Fos expression was significantly reversed by the vagotomy. ^*^ *P* < 0.05, significantly different between two groups of rats.

**Figure 7:**
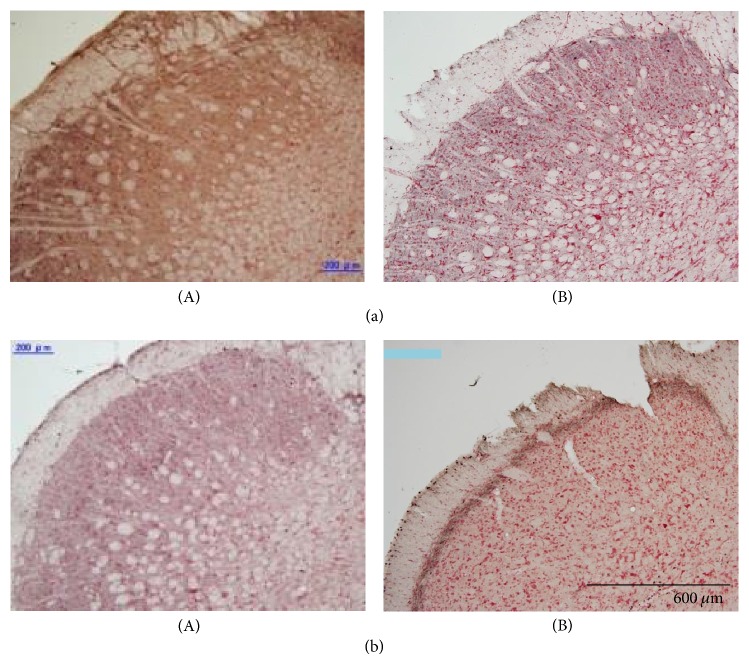
Effects of the lesion of the C1-C2 spinal segments on c-Fos expression in vehicle-infused (a) and PPS-infused (b) rats. Photomicrographs show c-Fos expression in the left Sp5C. Fos-labeled cells were observed in the intact rats (a-A and b-A). The number of Fos-labeled cells remarkably increased in the lesioned rats (a-B and b-B) compared with the intact rats.

**Figure 8:**
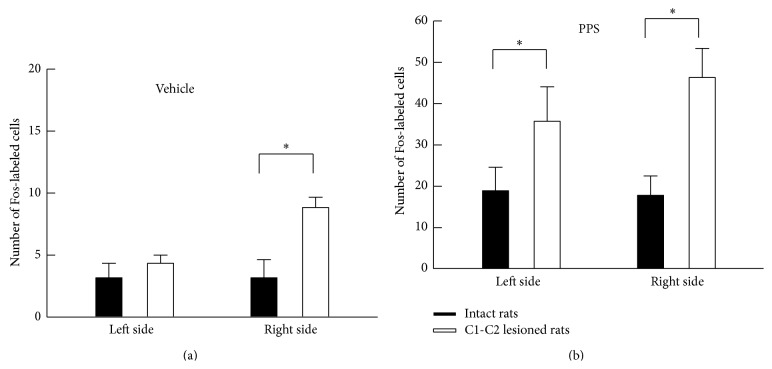
A comparison of the number of Fos-labeled cells between intact and C1-C2-lesioned rats following an infusion of either vehicle or PPS solution. Note that c-Fos expression significantly increased when the C1-C2 spinal segments were lesioned. ^*^ *P* < 0.05, significantly different between two groups of rats.

 For the PPS-infused rats (*n* = 6), the effects of the vagotomy on c-Fos expression in the C2 spinal segments and the Sp5C are shown in a representative example in Figure 5, and the number of Fos-labeled cells is compared between rats that did and did not receive the vagotomy in Figure 6. A bilateral section of the cervical vagus nerves showed a significant decrease in the number of Fos-labeled cells in both the Sp5C and C2 spinal segments. In particular, no Fos-labeled cells were observed in the Sp5C. In contrast, the lesion of the C1-C2 spinal segments produced a significant increase in the number of Fos-labeled cells in the Sp5C. This increase in the number of Fos-labeled cells was observed in both the PPS-infused (*n* = 6) and the vehicle-infused (*n* = 3) rats. Examples of c-Fos expression are shown in Figure 7, and the number of Fos-labeled cells is summarized in Figure 8.

## 4. Discussion

In the present study, special attention was given to the change of the mean blood pressure and the heart rate following both the infusion of either PPS solution or saline into the pericardial sac and the surgical treatments, which were either the vagotomy or the spinal cord lesion. The animal's mean blood pressure and heart rate did not change significantly compared to the values that were present before infusion or surgical treatments, although a slight decrease was observed. From these findings, the influence of the infusion-withdrawal procedure of either PPS solution or saline flush into the pericardial sac was excluded from the present study, as was the influence of surgical treatments. 

### 4.1. Involvement of the Sp5C in Angina-Induced Referred Pain in the Trigeminal Nerve Territory

 Some electrophysiological studies suggest that angina-induced referred pain in the trigeminal nerve territory is attributed to the convergence of both inputs from the trigeminal nerve territory and inputs from the cardiac region to the C1-C2 STT cells [4–8]. This convergence explained the mechanism that underlies referred pain in the trigeminal nerve territory associated with the angina pectoris. Clinical or experimental evidence of this effect has not yet been found. The present study was the first study to demonstrate that nociceptive signals from the cardiac region are input to the TSNC, specifically to the Sp5C. 

 To compare the number of Fos-labeled cells among the nuclei in the TSNC, we counted the total number of Fos-labeled cells in each nucleus, because each nucleus differed in area. Counting individual nuclei is considered an acceptable method for counting Fos-labeled cells in the trigeminal sensory nuclear complex [22–28]. In the present study, infusion of the PPS to the pericardial sac resulted in a significant increase in Fos-labeled cells in the Sp5C but not in other nuclei. The Sp5C is a component of the ascending sensory pathway that transmits nociceptive signals from the trigeminal nerve territory to the thalamus. Nociceptive information from the orofacial region is input to the Sp5C and then transmits to the ventral posteromedial (VPM) nucleus of the thalamus. Therefore, it is likely that the activity of the Sp5C neurons that is evoked by sensory signals from the cardiac region induces referred pain that is generally associated with an attack of angina pectoris in the orofacial region. Additionally, the unmyelinated afferent fibers that conduct nociceptive signals terminate to the superficial layers in the Sp5C [29]. The result in the present study that the number of Fos-labeled cells in superficial layers was significantly higher than that in the magnocellular layer suggests that the nociceptive signals from the cardiac region are transmitted to the thalamus via the superficial layers of the Sp5C. 

### 4.2. Peripheral Afferents Conducting Nociceptive Signals to the Sp5C

The heart receives reciprocal double innervations of the cardiac sympathetic and vagus nerves. Bilateral sections of the cervical vagus nerves completely reversed the c-Fos expression that was found in the Sp5C following the infusion of the PPS solution into the pericardial sac. This result shows that the nociceptive signals from the cardiac region to the Sp5C were conducted by the vagal afferent fibers. Additionally, a small amount of Fos-labeled cells was observed in the C2 spinal segment, which indicates that c-Fos expression may be induced by surgical procedures. 

Among the animals that received a lesion in the C1-C2 spinal segments, an increase in Fos-labeled cells in the Sp5C was observed in the vehicle-infused rats. This result suggests that the lesion of the C1-C2 spinal segments influenced c-Fos expression in the Sp5C. Therefore, it is apparent that the lesion procedures affected the increase in Fos-labeled cells in the PPS-infused rats. Unfortunately, we cannot explain this phenomenon in the present study. The rate of increase in the Fos-labeled cells was greater in the PPS-infused rats than that in the vehicle-infused rats. For the animals that received the C1-C2 lesion, the number of Fos-labeled cells was not reduced in the PPS-infused rats compared with the vehicle-induced rats; thus, it is possible that the nociceptive signals from the cardiac region were input to the Sp5C via a neural pathway in the brain but not via the spinal cord. Unfortunately, the location of this neural pathway was not identified in the present study. In humans, the vagal afferent signals from the visceral organs, including the cardiac region, are not input to the Sp5C, although the vagal afferent signals from the skin of the ear concha are input to the Sp5C [30]. Several morphological and electrophysiological studies have shown that vagal afferent fibers and the nucleus tractus solitarius play an important role in angina-induced referred pain in the trigeminal nerve territory [4, 5, 7, 14]. This evidence suggests that the nociceptive signals from the cardiac region are input to the Sp5C via the nucleus tractus solitarius. However, the pathway from the nucleus tractus solitarius to the Sp5C has not been confirmed in anatomical studies. It is possible that the output signals from the nucleus tractus solitarius are projected to the Sp5C by way of other nuclei in the brain. The existence of this possibility could not be clarified in the present study.

 The descending projection from the Sp5C to the spinal dorsal horn has been demonstrated anatomically [31–35]. Therefore, the activation of Sp5C cells induced by nociceptive signals from the cardiac region may explain the results from prior studies that injection of algesic chemicals into the pericardial sac resulted in an increase in activity among the C1-C2 STT cells [4–7]. 

In conclusion, nociceptive signals from the cardiac region are input to the SP5C via an unknown pathway in the brain. These signals are transmitted to secondary neurons at the superficial layers in the Sp5C and then projected to the thalamus. This ascending pathway may contribute to angina-induced referred pain in the trigeminal nerve territory.
